# Design, delivery and effectiveness of health practitioner regulation systems: an integrative review

**DOI:** 10.1186/s12960-023-00848-y

**Published:** 2023-09-04

**Authors:** Kathleen Leslie, Ivy Lynn Bourgeault, Anne-Louise Carlton, Madhan Balasubramanian, Raha Mirshahi, Stephanie D. Short, Jenny Carè, Giorgio Cometto, Vivian Lin

**Affiliations:** 1 Athabasca University, Athabasca, Canada; 2 University of Ottawa, Ottawa, Canada; 3 Royal Melbourne Institute of Technology (RMIT) University, Melbourne, Australia; 4 College of Business, Government and Law, Flinders University, Adelaide, Australia; 5 Menzies Centre for Health Policy and Economics, The University of Sydney, Sydney, Australia; 6 University of Sydney, Sydney, Australia; 7 University of Technology Sydney, Sydney, Australia; 8 World Health Organization, Geneva, Switzerland; 9 University of Hong Kong, Hong Kong, China; 10 Canadian Health Workforce Network, Ottawa, Canada

**Keywords:** Health practitioner regulation, Health systems, Health workforce, Systematic reviews, Integrative review

## Abstract

**Background:**

Health practitioner regulation (HPR) systems are increasingly recognized as playing an important role in supporting health workforce availability, accessibility, quality, and sustainability, while promoting patient safety. This review aimed to identify evidence on the design, delivery and effectiveness of HPR to inform policy decisions.

**Methods:**

We conducted an integrative analysis of literature published between 2010 and 2021. Fourteen databases were systematically searched, with data extracted and synthesized based on a modified Donabedian framework.

**Findings:**

This large-scale review synthesized evidence from a range of academic (n = 410) and grey literature (*n* = 426) relevant to HPR. We identified key themes and findings for a series of HPR topics organized according to our structures–processes–outcomes conceptual framework. Governance reforms in HPR are shifting towards multi-profession regulators, enhanced accountability, and risk-based approaches; however, comparisons between HPR models were complicated by a lack of a standardized HPR typology. HPR can support government workforce strategies, despite persisting challenges in cross-border recognition of qualifications and portability of registration. Scope of practice reform adapted to modern health systems can improve access and quality. Alternatives to statutory registration for lower-risk health occupations can improve services and protect the public, while standardized evaluation frameworks can aid regulatory strengthening. Knowledge gaps remain around the outcomes and effectiveness of HPR processes, including continuing professional development models, national licensing examinations, accreditation of health practitioner education programs, mandatory reporting obligations, remediation programs, and statutory registration of traditional and complementary medicine practitioners.

**Conclusion:**

We identified key themes, issues, and evidence gaps valuable for governments, regulators, and health system leaders. We also identified evidence base limitations that warrant caution when interpreting and generalizing the results across jurisdictions and professions. Themes and findings reflect interests and concerns in high-income Anglophone countries where most literature originated. Most studies were descriptive, resulting in a low certainty of evidence. To inform regulatory design and reform, research funders and governments should prioritize evidence on regulatory outcomes, including innovative approaches we identified in our review. Additionally, a systematic approach is needed to track and evaluate the impact of regulatory interventions and innovations on achieving health workforce and health systems goals.

**Supplementary Information:**

The online version contains supplementary material available at 10.1186/s12960-023-00848-y.

## Introduction

Health systems face considerable challenges in recruiting, training, distributing and retaining a sufficiently skilled and competent health workforce. These challenges are compounded by factors such as the increasing volume and privatization of health practitioner education, accelerating international mobility, a rise in cross-border service delivery; more team-based service delivery models, and the growing significance of frequently unregulated occupations like community health workers and traditional and complementary medicine (T&CM) practitioners [1].^1^

In response to the complex demands on health systems involving health workforces, some governments have reformed health practitioner regulation (HPR) systems to better serve the public interest [2–7]. HPR systems are increasingly acknowledged for their role in enhancing the availability, accessibility, quality, and sustainability of the health workforce, which is essential to make progress toward Universal Health Coverage and the Sustainable Development Goals [1]. Strengthening HPR systems can help to assure competence of the health workforce and the safety of services they provide, and foster the flexibility and innovation needed to meet population health needs. HPR can maximize the potential of the existing health workforce and assist in aligning health workforce investments with health system needs [8, 9].

There are significant gaps in our knowledge about leading HPR policy and practice, such as which regulatory models, institutional governance and combination of regulatory functions work best in different contexts. This review aimed to synthesize the evidence base around HPR design and delivery to help governments, regulators, and policymakers achieve health system and workforce goals.

### Defining health practitioner regulation

Based on the International Standard Classification of Occupations [10], we defined health practitioners to include health professionals, associate health professionals, and personal care workers in health services. We excluded categories of health workers not directly engaged with patient care or diagnostics, such as health care management and support staff. Practitioners from all areas of practice (acute, home, community, or public health) were included if they fit within the definitions of this classification (e.g., public health nurses were included while chief public health officers were not).

We use the term HPR to describe occupational regulation targeted at health practitioners; that is, the legally defined requirements or rules that govern entry into health occupations and subsequent conduct within those occupations [11]. The term HPR encompasses a jurisdiction’s suite of laws, regulations, bylaws, decrees, codes, directives, or other rules targeted explicitly at health occupations. While HPR may be defined broadly to include occupational rules set by various bodies such as non-governmental or self-regulatory bodies [12], this review primarily focuses on the rules established by governments or professional bodies operating under government delegation or recognition.

We use statutory registration as an umbrella term that captures schemes that apply either or both reservation of title (sometimes referred to as “registration”) and reservation of practice (sometimes referred to as “licensing”). When referring to statutory registration, we exclude certification, co-regulation, negative licensing or any other occupational regulation scheme.

These schemes can function either in conjunction with statutory registration or in its absence, depending on the country context and occupation.

### Guiding conceptual framework

**Fig. 1:**
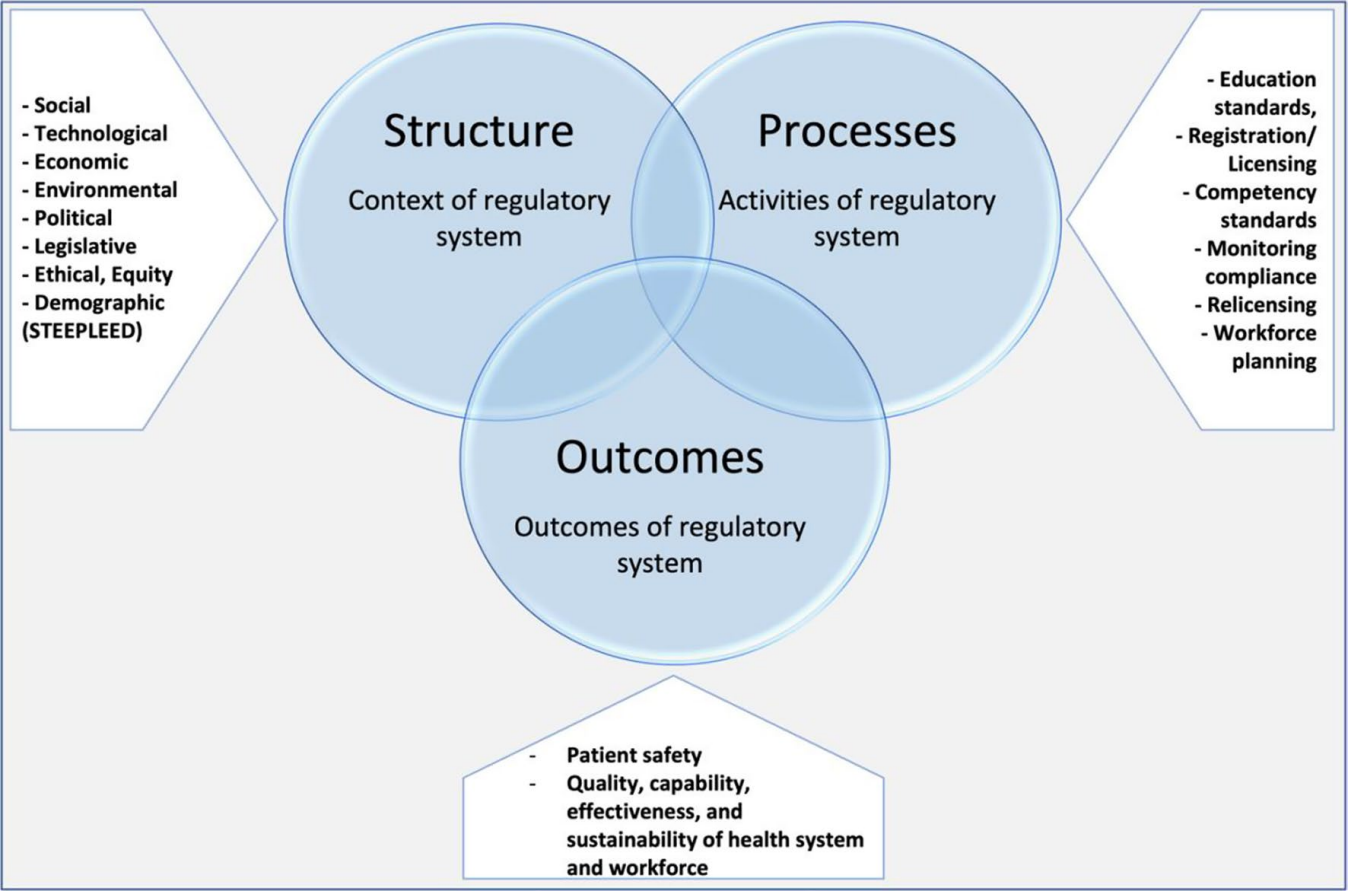
Modified Donabedian framework of HPR systems

We developed a modified Donabedian conceptual framework to guide this review (Fig. 1). *Structures* represent the context of HPR systems and include social, technological, economic, environmental, political, legislative, ethical, equity and demographic country/regional contexts (a modified STEEPLED framework, adding an equity dimension). *Processes* include the functions and activities of the HPR system, which may include, for example, setting qualification requirements for entry to practice, registering qualified practitioners, maintaining a public register, setting practice standards, monitoring continuing competence, managing complaints and fitness to practice proceedings, prosecuting offences, and supporting government health workforce planning and health system improvement. These processes are based on the analytical framework for understanding regulatory functions set out in the WHO’s *Western Pacific Regional Action Agenda on Regulatory strengthening and convergence for medicines and health workforce* [9]*. Outcomes* encompass various parameters such as the safety, quality and effectiveness of the workforce, the efficiency and effectiveness of a regulator or regulatory system in achieving its mandate and its contribution to achieving broader health system goals and priorities.

### Review question

This review was guided by an overarching question:

What key considerations, common principles, core elements, and recent innovations can assist jurisdictions in designing and delivering more effective HPR to improve patient safety and the quality, capability, effectiveness, and sustainability of their health workforce and achieve health system goals?

From this question, we developed a series of operational questions across the three elements of the conceptual framework to guide the search, selection and synthesis of evidence (Table 1).

**Table 1:** Operational questions based on the conceptual framework

Elements of conceptual framework	Operational questions
Structures: Context of HPR systems	What contextual forces and structural characteristics shape the design and delivery of HPR functions, and what are the key challenges governments and regulators face?
Processes: Functions and activities of HPR systems	What are the main functions and activities of HPR systems, and what diversity of approaches, models and tools are evident in how these functions are organized and delivered?
Outcomes: Impact and effectiveness of HPR systems and processes	How effective are various approaches and models of HPR in improving the safety, quality, quantity, capability, and effectiveness of health systems and workforces?

## Review method

We used a rapid review methodology [13, 14] for this large-scale integrative review. Applying rapid review methods was a pragmatic choice due to the lack of common HPR terminology and the need to capture a range of evidence (sources and types) from many disciplines and jurisdictions to answer the overarching and operational research questions [15–17]. The research design accommodated these various contexts and perspectives, providing the opportunity to examine a range of evidence (arising from qualitative, quantitative, correlation, economic, policy, regulatory, and other sources) to summarize the global literature on HPR at practitioner, organizational, and societal levels [15–17].

Due to the topic and the breadth of the multidisciplinary academic and grey literature reviewed, we did not conduct risk of bias or formal certainty of evidence assessments on the included studies. We did not apply the GRADE (Grading of Recommendations, Assessment, Development and Evaluation) framework since most of the literature included was descriptive or observational and thus would have been classified as very low or low certainty, despite the valuable insights offered by this literature. Further, the factors that can increase the certainty of evidence under GRADE (large magnitude of effect, dose–response gradient, and effect of plausible residual confounding) have little applicability when reviewing descriptive or observational studies, such as those identified on HPR through this review. The nature of the available literature pointed to a broad assessment of very low certainty of the evidence. Further information on the research design, including a diagram of our design, example database searches, and a modified PICO framework, are available in Additional file 1.^2^

### Search strategy

The multidisciplinary nature of the literature on HPR and the broad research question required us to set wide parameters for the search strategy and adopt an interdisciplinary approach. An iterative three-step search strategy was employed using specific keyword searches developed in consultation with librarians and subject experts in regulation, health policy, sociology, economics, law and public health and revisited as useful search terms were discovered and employed [18, 19].

First, an initial limited search was conducted in Scopus and EMBASE. Using the results of this search, the research team analyzed text in the title, abstract, keywords, and index terms used to describe the retrieved articles. Second, this analysis was used to create a revised search strategy that we extended across academic databases, including Medline, Embase, Web of Science, Cochrane Library, CINAHL, PsycINFO, PsychARTICLES, Scopus, Sociological Abstracts, ProQuest Dissertations and Theses Global, and JBI EBP. Specialist databases including HeinOnline, World Legal Information Institute (WLII) and the ILO Legal Database were also searched. We conducted hand searches on Google and TRIP Clinical search engines. National online legislative databases were used to identify relevant extant legislation. Finally, to ensure literature saturation, we also used citation tracking and forward–backward searches of references in the included articles, reports and policy documents. The WHO Technical Expert Group on HPR identified additional sources for screening throughout the review process.

### Eligibility criteria

Sources were selected for inclusion if they described a HPR legislative instrument, regulatory system, regulator or regulatory function or intervention, or if they examined factors shaping the development, operation, or outcomes of HPR in terms of health systems or workforce goals. Grey literature included reports from international organizations, HPR consortia, regulators and meta-regulatory bodies, and government and intergovernmental policy documents that discussed HPR systems of one or more jurisdictions. Sources published from 2010 to 2021 in English, French, Spanish, Portuguese and Chinese were eligible for inclusion. Older references (before 2010) identified via citation tracking or by our expert advisors were included if directly applicable to our research question.

We included both qualitative and quantitative research. Original research articles and reviews were included from the academic literature. Commentaries, policy papers and perspectives were included where they provided substantive content or critique of HPR-related contexts, performance or reform directions. We included government reports, statutes, and policy documents from the grey literature that examined the HPR systems of one or more countries. Review management software Covidence [20] was used to screen articles and select published articles for extraction by two reviewers, with a third reviewer assessing conflicts.

### Data extraction and synthesis

Data extraction from included articles used Covidence (for academic articles) and Excel (for grey literature). Data extraction was based on a predefined tool to categorize articles by two dimensions:

1. Sources were classified according to a predetermined set of general topic areas and organized according to structures, processes and outcomes. Themes within these topic areas were identified and tracked.
2. Data were extracted using a modified PICO framework (Population/Practitioner, Intervention/HPR Approach, Context/Country and Outcome) for synthesis in tabular format.

We used Sandelowski’s ‘integrated synthesis’ approach for synthesizing the qualitative and quantitative evidence [18, 21]. Under this framework, both forms of data (quantitative and qualitative) are combined through a single mixed-methods synthesis approach, with assimilation achieved by converting quantitative data into themes that are codified and presented along with qualitative data in a narrative or aggregated format.

## Findings

**Fig. 2:**
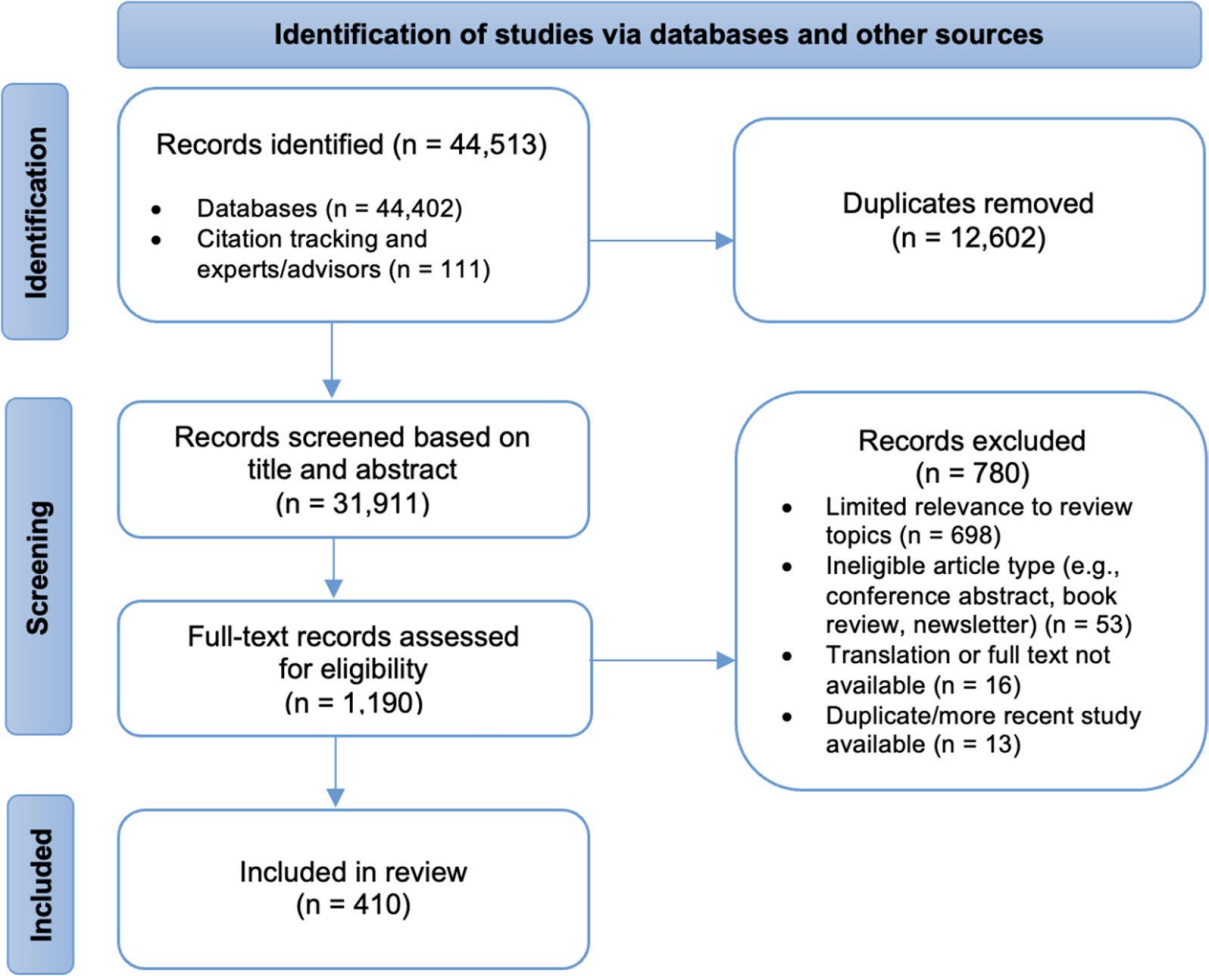
PRISMA flow diagram for academic literature sources

We included 410 academic articles and 426 grey literature sources in the review. Not all sources are referenced in this article because we synthesized the main thematic findings and prioritized references accordingly. A description of all sources with reference and selected extraction data is available in an additional spreadsheet file (see Additional file 3). Figure 2 illustrates the PRISMA flow diagram for the academic literature sources (*n* = 410) included in the review [22].

**Fig. 3:**
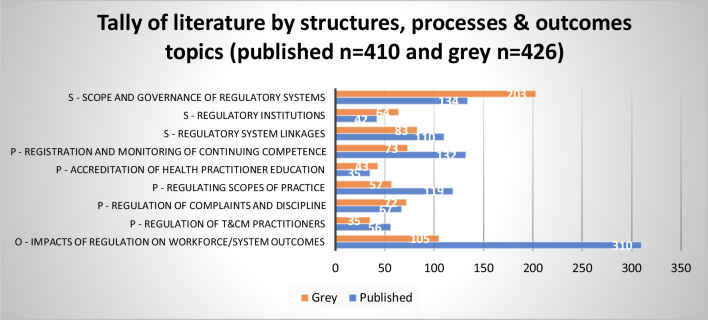
Distribution of published literature by topic and structures (S), processes (P), and outcomes (O)

**Fig. 4:**
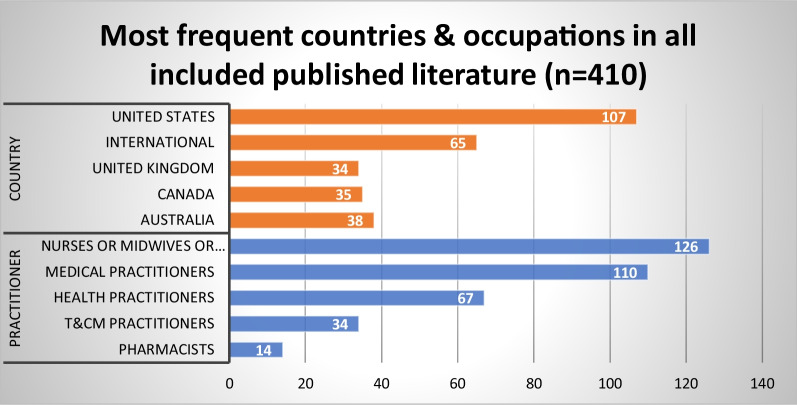
Most frequent countries and health occupations in the published literature (*n* = 410)

**Fig. 5:**
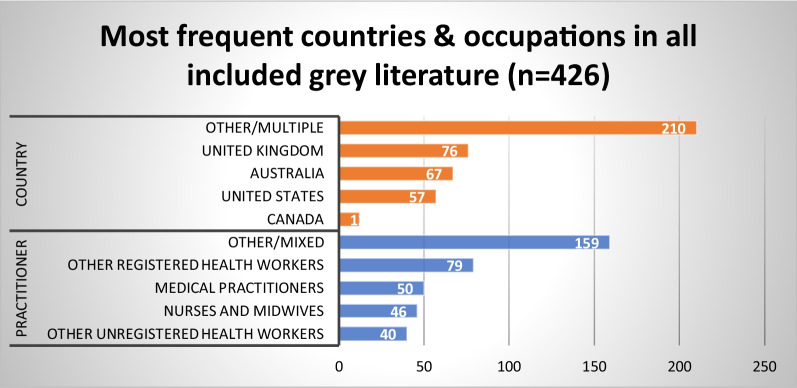
Most frequent countries and health occupations in the grey literature (*n* = 426)

Figures 3, 4, and 5 provide an overview of evidence sources for each topic, organized according to structures, processes and outcomes, and the predominant countries and health occupations studied in the published and grey literature. Further details on the countries and health occupations in the academic sources are available in Additional file 2.

According to the integrative review approach adopted, the topics were identified as part of the review process and using the modified Donabedian framework as follows: (A) structures (including scope and governance of regulatory systems, institutions and system linkages); (B) processes (including registration and monitoring of continuing competence, accreditation of health practitioner education, regulating scopes of practice, management of complaints and disciplinary matters, and regulation of T&CM practitioners); and (C) outcomes (impacts of regulation on health workforce and system outcomes).

**Fig. 6:**
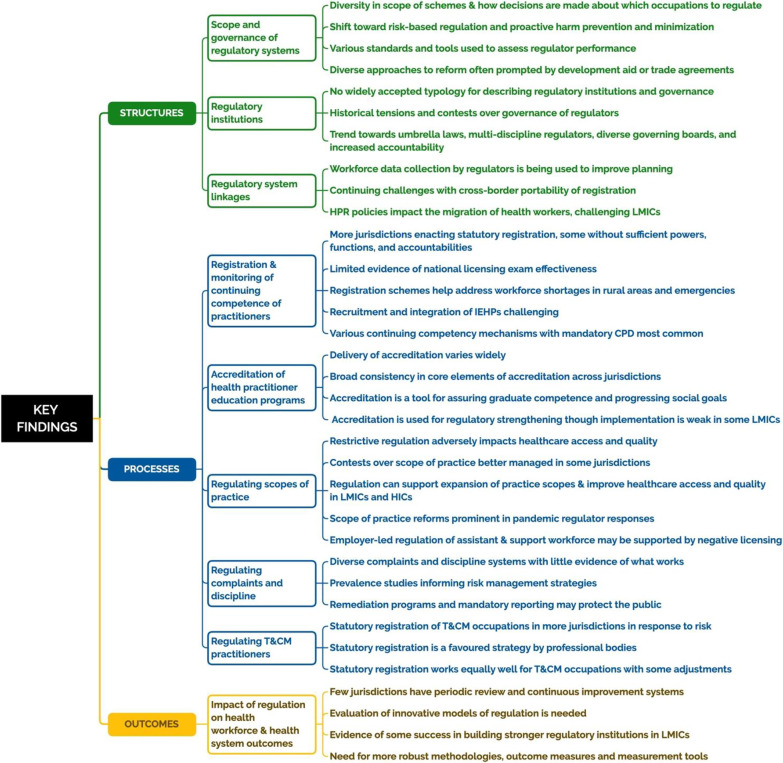
Overview of topics and themes categorized by structures, processes, and outcomes

We identified key themes based on our integrated synthesis of the data, clustered under a series of HPR topics organized according to our structures–processes–outcomes conceptual framework. These HPR topics and themes are summarized in Fig. 6 and key messages are outlined in Table 2.

**Table 2:** Key messages from this review categorized by structures, processes, and outcomes

Topic	Key messages
Structures: Scope and governance	Governance reforms show a trend toward umbrella laws, multi-profession regulators, more diverse governing boards, and increasing accountability and oversight measures Increasing reliance on principles and tools of risk-based regulation signals a shift to more proactive strategies for harm prevention and mitigation
Structures: Institutions	Most studies focused on statutory registration, a model increasingly being used across various jurisdictions and practitioner groups The lack of standardized HPR typology complicates comparisons and makes it difficult to draw conclusions about the effectiveness of various governance models
Structures: System linkages	HPR can support government strategies for workforce planning, development, supply and distribution, particularly to address workforce shortages in rural areas and during emergencies Despite efforts in harmonization and mutual recognition, challenges remain with cross-border recognition of qualifications and portability of registration, impacting health worker migration and mobility
Processes: Scopes of practice	Scope of practice regulation can adapt to health system demands for collaborative team-based practice and a more dynamic division of labor Scope of practice reforms, particularly around prescribing rights for non-physician clinicians, can improve healthcare access and quality
Processes: Continuing competence	Outcomes-based CPD models can be effective continuing competence mechanisms if access, equity, delivery, and design are addressed Programs that support internationally educated health practitioners can aid recruitment and successful transition to practice
Processes: Accreditation of health practitioner education programs	Core elements of accreditation are broadly consistent across jurisdictions and there is a growing presence of international accreditation agencies and standards Despite a lack of evidence on outcomes or cost-effectiveness, accreditation is considered important for assuring graduate competence in many jurisdictions and is a focus for regulatory strengthening initiatives in LMICs
Processes: Complaints and discipline	Remediation programs to support safe return to practice and clear mandatory reporting obligations can be effective public protection mechanisms
Processes: T&CM practitioners	Statutory registration can strengthen public protection for T&CM occupations based on risk profiles and is increasingly used to preserve indigenous medical knowledge and improve health service delivery to underserved populations
Outcomes	Alternatives to statutory registration for lower-risk health occupations can improve health services and consumer protection A broader systems approach to evaluating regulatory failures and standardized evaluation frameworks can aid regulatory strengthening initiatives

### Structures

#### Scope and governance of regulatory systems

**Fig. 7:**
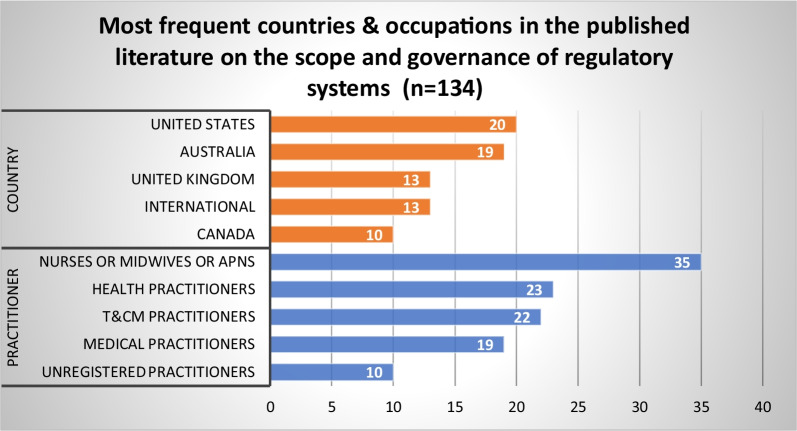
Most frequent countries and health occupations in published literature addressing the scope and governance of regulatory systems

A total of 134 published articles and 203 grey literature sources addressed HPR governance systems. Published articles primarily focused on nurses, midwives and advanced practice nursing (APN) roles (*n* = 35), followed by other health practitioners (*n* = 23) and T&CM practitioners (*n* = 22) (Fig. 7). Most literature came from the United States (US) (*n* = 20), Australia (*n* = 19) and the United Kingdom (UK) (*n* = 13). Four themes were identified from our synthesis of the published and grey literature on this topic.

*First,* there is diversity in the purpose, scope and features of regulatory systems and how decisions are made about which health occupations should be regulated.

Shaped by contextual factors such as the historical division of labor and population health needs [23–25], there is jurisdictional variation between which occupational groups are regulated and how. While most jurisdictions have some form of legislated licensing scheme for one or more health occupations, the purpose, scope and features vary. More jurisdictions are using the principles of good regulatory practice to strengthen the evidence base for these contested decisions [26–31]. The literature suggests that in jurisdictions without strong regulatory management systems, some occupational groups are being licensed when a less resource-intensive type of occupational regulation may provide sufficient public protection at a lesser cost to the practitioner, the regulator and the community [5, 28, 32–36].

*Second,* the principles and tools of risk-based regulation adopted by some regulators signal a shift to more proactive strategies for harm prevention and minimization.

The literature describes how regulators use data analytics tools to refocus regulatory resources, systematically identifying ‘hotspots’ of risk (due to registrant competence or conduct issues) and developing targeted harm reduction programs [37–42]. Some literature suggests that risk-based regulatory strategies have been applied more widely during the COVID-19 pandemic—more nimble regulators have weighed the risks and benefits to the public of various regulatory actions used to facilitate a surge workforce [43–48].

*Third*, various generic and HPR-specific standards and tools are being used to assess HPR performance, with some adaptable for use in lower-resource environments.

The literature presents a range of frameworks and tools used by governments to improve regulatory policy and practice, from generic whole-of-government good regulatory practice frameworks [28–31] to HPR-specific evaluation tools [36, 40, 49–52]. We identified an increased focus in the grey literature from high-income countries (HICs) on assessment and accountability standards that apply to regulators, including heightened scrutiny of regulatory operations by integrity agencies and other independent review bodies [2, 53–57].

*Fourth,* there are diverse approaches to regulatory reform, with studies reporting new regulation or regulatory strengthening activities in LMICs, sometimes prompted by development aid or trade agreements.

Jurisdictional regulatory reform processes range from successive system-wide reviews and ongoing formalized reform programs [2–4, 6, 7, 53, 55, 58, 59] to more incremental, piecemeal or ad hoc reforms [23, 34, 60, 61]. In LMICs, studies documented the establishment of new regulators and other regulatory strengthening initiatives, sometimes associated with development funding. Six studies from sub-Saharan African countries presented results from the African Health Profession Regulatory Collaborative [62–67]. They reported substantial and sustainable advances in regulating nurses and midwives in Africa, offering a framework for evaluating future progress. In Europe and South-East Asia, studies referred to the role of trade agreement mutual recognition arrangements in motivating governments to establish or reform licensing schemes [68–77].

#### Regulatory institutions

**Fig. 8:**
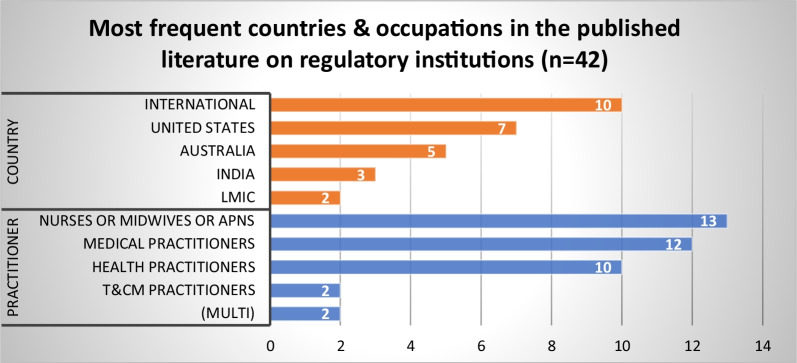
Most frequent countries and health occupations in the published literature on regulatory institutions

Our review identified 42 published articles and 64 grey literature sources addressing the institutional arrangements under which HPR functions are delivered. The published literature was primarily on nurses and midwives (*n* = 13), followed by medical practitioners (*n* = 12) and health practitioners in general (*n* = 12). International (global and multi-country) studies were prominent (*n* = 10),^3^ followed by studies from the US (*n* = 7) and Australia (*n* = 5) (Fig. 8). Three themes were identified from the synthesis of the literature on this topic.

*First,* there is no widely accepted typology for describing HPR institutional and governance arrangements.

There is considerable diversity in the institutions responsible for HPR and their governance arrangements, reflecting diverse political, social, and professional contexts [23, 24, 78–83]. Much of the published literature compared the governance arrangements of regulators across multiple jurisdictions [5, 23, 24, 78, 80, 83–95] or analyzed the strengths and limitations of specific elements of governance [85, 96–99]. There was no widely accepted or commonly used taxonomy for describing the features of HPR institutions, and terms such as ‘independent’, ‘autonomous’, ‘profession-led’ and ‘government-led’ were used without clear or standardized operational definitions.

*Second,* tensions between ‘profession-led’ governance models and increasing government expectations for oversight and control of regulators reflect a long history of contestation in some jurisdictions over who controls the institutions that govern health practitioners.

Some researchers highlighted the potential for conflicts of interest where the regulator operates within a health ministry with broader service delivery and stewardship responsibilities, calling for reforms to strengthen the independence of regulators from governments [80, 85, 86, 100]. Similarly, some international professional associations argue for ‘profession-led’ (or ‘professional self-regulation’) rather than government-led regulation [101–103]. Conversely, other sources questioned governance arrangements where the regulator is constituted with elected members of the occupational group being regulated, with calls to reduce the level of control exercised by health practitioners and increase government oversight [2–4, 6, 58, 84]. A shift away from governance models that embed ‘representativeness’ (of those being regulated) and towards greater government oversight and control is evident primarily in Anglophone countries with a long history of delegating regulatory powers to ‘profession-led’ bodies. The grey literature suggests that governments are placing greater expectations on regulators to be more transparent and accountable in their operations, better manage conflicts of interest (through, for example, structural separation of investigation functions from determinative functions in disciplinary matters) and ensure registrants are afforded procedural fairness [2, 3, 6, 53, 54, 95, 104–106].

*Third,* HPR governance reforms show a trend toward umbrella laws, multi-profession regulators, more diverse governing boards and increased accountability obligations.

There is evidence of trends toward the use of umbrella statutes and multi-profession regulatory agencies, with studies from LMICs and HICs suggesting considerable net benefits [24, 37, 68, 95]. There is some evidence from HICs that, by achieving greater economies of scale, multi-profession regulators might be more efficient than large numbers of small profession-specific agencies [55, 107]. WHO publications and government reviews have encouraged multi-profession governance to address the disadvantages of profession-specific regulatory ‘silos’ for setting education and practice standards and administering disciplinary and enforcement functions [2, 9, 53, 108]. These models also enable more efficient updating of the legislative framework and facilitate international collaboration [6, 109].

#### Regulatory system linkages

**Fig. 9:**
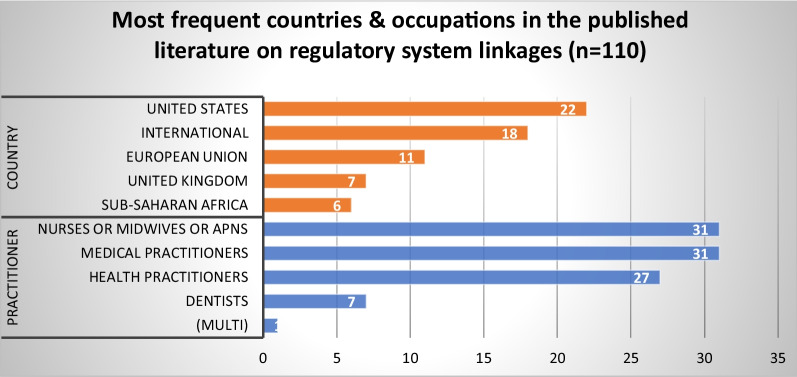
Most frequent countries and health occupations in the published literature on regulatory system linkages

Our review examined evidence concerning the nature of the interfaces and linkages between HPR and other quality assurance mechanisms, within health systems and with other institutions and sectors beyond health. This literature included 110 published articles and 83 grey literature sources. The published articles focused primarily on nurses and midwives (*n* = 31) and medical practitioners (*n* = 31), followed by health practitioners generally (*n* = 27) (Fig. 9). Articles came primarily from the US (*n* = 22), followed by studies with a global or international focus (*n* = 18) and Europe (*n* = 11). Two themes were identified from our synthesis of the published and grey literature.

*First,* routine collection by regulators of comprehensive workforce data is being used to improve health workforce planning, development, supply and distribution.

The literature shows how HPR can directly impact workforce supply and facilitate (or hinder) a flexible, responsive, and sustainable health workforce [1, 4, 6, 108]. The literature also reveals an increasing recognition of the role of regulators in collecting and supplying to governments registrant data for use in health workforce planning [110–112]. Several reports highlighted how the COVID-19 pandemic has rapidly escalated the need for timely workforce data collection, planning and mobility [113–115]. Actions taken by regulators to support a surge workforce during the pandemic were highlighted, including widespread scope of practice reforms, fast-tracked licensing and foreign credential recognition, rapid recruitment from abroad and from final year medical and nursing students, rapid retraining using online learning, incentivizing labor mobility, and setting practice standards and guidelines to support the delivery of virtual care [43, 46–48, 114, 116–118].

*Second,* despite continuing efforts for harmonization and mutual recognition, challenges remain with cross-border recognition of qualifications and portability of registration.

Many studies addressed the challenges faced by regulators in responding to the demand for greater mobility of health practitioners across jurisdictions, including under mutual recognition arrangements.^4^ These challenges relate to factors such as the variability in requirements for registration (e.g., qualifications, examinations), the diversity of requirements for renewal of registration (e.g., CPD, revalidation), the need to assure the competency of practitioners providing virtual care, and the management of disciplinary matters that require regulators to share information or that raise cross-border jurisdictional issues [25, 72, 77, 119–122].

*Third,* HPR policies impact the migration of health workers.

Studies point to the role of HPR policies (e.g., qualifications required for entry, local language requirements, types of registration available) in contributing to international migratory flows of skilled health personnel. Several studies noted the challenges with the implementation of the 2010 *WHO Code of Practice on International Recruitment of Health Personnel* [123–128]. A complex range of push and pull factors were identified, with gaps in knowledge about the effectiveness of policy interventions that might regulate the movement of health practitioners from LMICs to protect vulnerable health systems, particularly in times of medical emergency.

### Processes

#### Registration and monitoring of continuing competence of practitioners

**Fig. 10:**
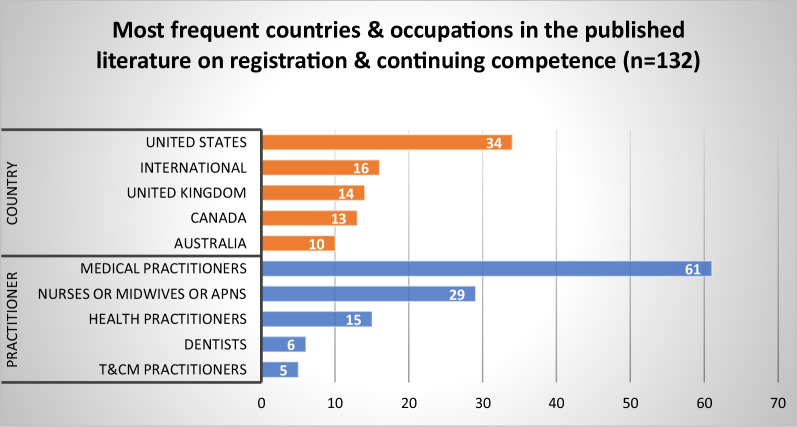
Most frequent countries and health occupations in the published literature on registration and monitoring of continuing competence of practitioners

We examined the literature on HPR registration processes, including setting standards for registration, processing applications, monitoring standards of practice and the continuing competence of registrants, and the operation of public registers. We identified 132 published articles and 73 grey literature sources (Fig. 10). Most published articles came from the US (*n* = 34), followed by international studies (*n* = 16), and the UK (*n* = 14). Articles focused primarily on medical practitioners (*n* = 61), nurses, midwives and APN roles (*n* = 29), and health practitioners generally (*n* = 15). Five themes were identified from our synthesis of the published and grey literature.

*First*, while there are signs of regulatory convergence as more jurisdictions establish statutory registration schemes, some schemes lack a comprehensive set of powers, functions, and accountabilities.

There is evidence that many LMICs and HICs with differing legal traditions have enacted statutory registration schemes for key occupational groups, such as medical doctors, nurses, midwives, dentists and pharmacists.^5^ In some cases, regulators may lack a comprehensive suite of necessary powers, functions and accountabilities. For example, they might not have the authority to grant different types of registration, mandate annual registration renewals, monitor compliance with practice standards, or enforce disciplinary actions for violations; similarly, they may not be obliged to ensure procedural fairness in regulatory decision-making, collect and provide practitioner data for workforce planning and system improvement, or routinely report on the performance and outcomes of regulatory activities [9, 23, 78, 80, 81, 88, 89, 108, 129, 130].

*Second,* the evidence on the effectiveness of the national licensing examination (NLE)^6^ for assuring graduate capability is limited, and the complexities of running a robust and reliable NLE can be underestimated.

Four UK-authored systematic reviews examined whether NLEs assure practitioner competence or improve patient safety and found the evidence was weak [131–134]. Several studies from LMICs highlighted factors contributing to the pressure to introduce an NLE, such as the rise in private sector education providers resulting in a surplus of graduates and uncertain standards, the need to standardize training and entry to the public service, and to improve quality of care [71, 77, 135–138]. These studies also highlighted the complexities of introducing NLEs, including in the context of mutual recognition agreements that seek to harmonize entry requirements to promote fairness, the common market, and freedom of movement [77].

*Third*, statutory registration schemes can help governments address workforce shortages in rural areas and during emergencies.

The literature discussed the role of HPR processes in addressing the challenges of securing a sufficient rural workforce in LMICs [70, 139–142] and HICs [143–147]. Regulatory tools can support the implementation of broader rural workforce recruitment, retention and development strategies. Examples include compulsory service requirements tied to registration or modified qualification requirements, scopes of practice and supervision arrangements for practitioners recruited specifically to work in areas of workforce shortage [70, 139–142]. In HICs, the literature focused on regulatory changes made or advocated to support advanced practice nurses serving rural communities. There is substantial evidence that jurisdictions enabling autonomous advanced nursing practice achieve a higher supply of these nurses, improve patient access to health services, and better healthcare outcomes, especially in rural and underserved areas [143, 144, 148].

*Fourth*, recruiting and integrating internationally educated health practitioners into the local workforce present particular challenges, with some evidence of effective integration programs.

Studies examined how statutory registration impacts internationally educated health practitioners (IEHPs), focusing on how well they integrate into the local health workforce. Studies evaluated the impact of assessment requirements [149–153], comparative rates of disciplinary or fitness to practice actions against internationally and locally educated practitioners [154, 155], the implementation and effectiveness of specific transition-assistance programs [146, 156] and the broader implications of IEHP mobility [157, 158], mainly from the point of view of destination countries. Various international conventions, treaties and intergovernmental trade agreements were instrumental in encouraging governments to remove or reduce barriers and facilitate health practitioner mobility [159–165].

*Fifth*, while regulator-mandated continuing professional development (CPD) is common and can be effective, various continuing competency mechanisms are found in HICs, with limited evidence of comparative effectiveness.

Continuing competency mechanisms vary across jurisdictions and practitioner groups in the same jurisdiction. These mechanisms include mandatory CPD standards required to renew registration [166–168], certification and recertification programs run by a range of non-government bodies [122, 169–171], maintenance of certification programs run by specialist colleges [172–177], and revalidation programs run in partnership between regulators and employers [131, 178, 179]. Requiring participation in CPD is the most common mechanism used by regulators to assure the continuing competence of registrants. Some studies point to deficiencies in these requirements where insufficient attention is given to the context, the learner’s needs and the delivery methods [180–183]. Evidence suggests a link between CPD requirements and improved skills and knowledge [184, 185]. In LMICs, mandatory CPD linked to registration can be a pivotal strategy to lift the skills of various health workers, but adequate enforcement and continued resource inputs are required [62, 71, 186–189].

#### Accreditation of health practitioner education (HPE) programs

**Fig. 11:**
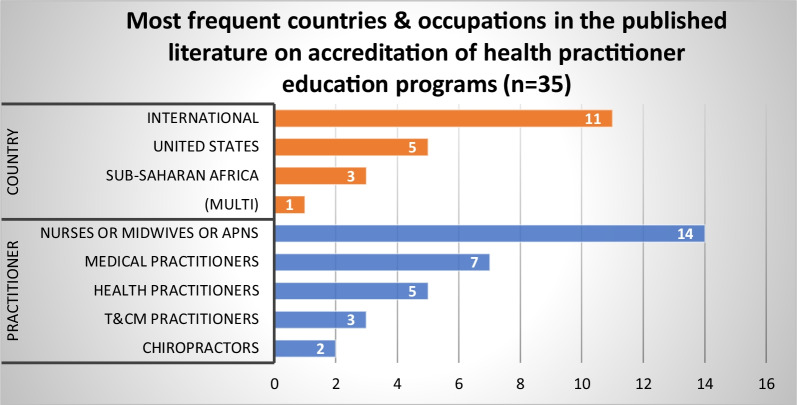
Most frequent countries and health occupations in published literature addressing accreditation of HPE programs

Literature on the role of HPR in accrediting education programs for entry to practice included 35 published articles and 43 grey literature sources. The published literature on this topic focused primarily on nurses and midwives (*n* = 14), followed by medical practitioners (*n* = 7) and health practitioners generally (*n* = 5) (Fig. 11). The international literature was most prominent (*n* = 11), followed by articles on the US (*n* = 5) and sub-Saharan Africa (*n* = 3). Four themes were identified from our synthesis of the published and grey literature.

*First,* arrangements for delivering HPE accreditation for entry-to-practice programs vary across jurisdictions and occupations.

The responsibility for evaluating and assuring the quality of HPE programs, and the governance arrangements under which they operate, differ across and within various jurisdictions. This function may be carried out by one or more statutory regulators, the responsible education ministry, or a non-government professional body under delegation from government. Sometimes there is an oversight body that brings together key government, regulator and non-government entities. This diversity extends to the linkages between the health and education sector accreditation processes (if any), the extent of coverage of public and private sector institutions and programs, and the transparency of operation and performance of accreditation systems [5, 24, 25, 68, 82, 94, 95, 190–194]. In some jurisdictions, graduation from a program of study accredited by the regulator is sufficient to qualify for registration [106, 195]. In others, graduates of accredited programs must also sit an NLE [5, 94, 95]. Several reports highlighted the interdependence of the health and education sectors in quality assuring HPE programs and the need for stronger coordination and joint standard setting [106, 196]. No studies were identified that evaluated the effectiveness of different governance models.

*Second,* despite the diversity in governance, core elements of HPE accreditation appear broadly consistent across jurisdictions and there seems to be a growing involvement of international accreditation agencies and standards.

While several studies noted a lack of evidence to support accreditation as a tool for quality assuring the health workforce [197–199], this review found broadly similar core elements of HPE accreditation described in the literature [199–202]. Also evident is a shift to outcomes-based measures and competency-based education [50, 197, 200], including in documents published by international standard-setting bodies such as the International Confederation of Midwives and the World Federation of Medical Education [203, 204].

*Third,* while there is little evidence of the effectiveness of HPE accreditation, it is considered an important tool for assuring graduate competence for entry-to-practice and progressing broader social goals.

The review found little published literature assessing the effectiveness of HPE accreditation in producing skilled and competent practitioners [197, 198]. No studies were found that compared jurisdictions with and without HPE accreditation or compared HPE accreditation with other quality assurance mechanisms such as national examinations. Despite the limited evidence base, some have pointed to the potential to use accreditation to achieve broader societal goals, such as increasing equity, diversity and cultural sensitivity of the workforce and removing racial discrimination from the health system [196, 205].

*Fourth,* HPE accreditation is being used as a tool for regulatory strengthening, although implementation is often weak, especially in some LMICs.

There is evidence that establishing HPE accreditation in LMICs has been prioritized in regulatory strengthening programs, particularly for nurses and midwives. The largest group of studies was associated with regulatory strengthening programs in sub-Saharan African countries [66, 135, 186, 191, 194, 206, 207]. There were also studies from Cambodia, India, Nepal and Vietnam [68, 208, 209]. Initiatives to introduce or strengthen accreditation of education programs and institutions were embedded within broader HPR reform programs designed to improve the quality of the health workforce [66, 68, 191, 206]. There is, however, some evidence in the grey literature that the implementation of accreditation standards in some LMICs is hampered by insufficient mechanisms to ensure compliance [92–94, 192].

#### Regulation of scopes of practice

**Fig. 12:**
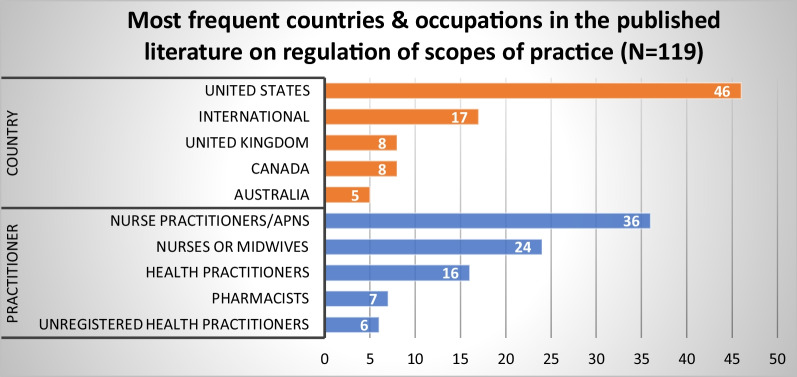
Most frequent countries and health occupations in published literature addressing regulation of scopes of practice

We examined the literature on different approaches to regulating practitioner scopes of practice and their impact on health workforce capability, flexibility and patient access to safe, high-quality services. The 119 published articles and 57 grey literature sources on this topic predominantly focused on nurse practitioners or APN roles (*n* = 36), nurses or midwives (*n* = 24), followed by health practitioners generally (*n* = 16) (Fig. 12). The US was the most studied country (*n* = 46), followed by international studies (*n* = 17) and Canada or Australia (*n* = 8 each). Four themes were identified from our synthesis of the published and grey literature.

*First*, there is evidence that restrictive and unresponsive scope of practice regulation is stifling innovation, inhibiting workforce reform and adversely impacting healthcare access and quality.

The literature shows how regulators in some jurisdictions are empowered to use reserved practice provisions to control which occupations or classes of registrants may carry out certain procedures and who must work under supervision or only on referral. Such blanket occupation-based and centrally administered restrictions may hamper the development of team-based care and other innovative models of care, and many studies documented the adverse impacts on access to and quality of care [144, 148, 210–220]. Overly restrictive scopes of practice were criticized during the COVID-19 pandemic, with both published and grey literature documenting the need for more flexibility in determining local health service roles and skill mix and enabling task shifting to support the crisis response [221–224].

*Second,* conflicts over scopes of practice reflect the tensions and competing interests between occupations.

The literature on scope of practice reform underlines the complexities of a dynamic and evolving division of labor in the health sector, the modern context of team-based and collaborative practice, and the urgency of workforce reform to improve access to care. Comparative studies emphasize the need to use the best available evidence to inform scope of practice reform [43, 225–228] and grey literature sources propose criteria and processes to strengthen evidence-informed decision-making and better manage competing interests and politics [5, 229–234].

*Third*, using HPR to support expanded scopes of practice, such as authorization to prescribe or administer restricted medicines, is improving healthcare access and quality in LMICs and HICs.

There is evidence that expanding scopes of practice to encompass prescribing and administering restricted medicines improves access to and quality of care, particularly for rural or other underserved populations [98, 212, 235–249]. The role of regulators includes setting the necessary competencies, accrediting training programs, monitoring compliance with standards for safe use of medicines and dealing with registrants breaching accepted practice standards [6, 250].

*Fourth*, with increasing reliance on health associate professionals, quality assurance of this workforce relies primarily on employer measures, although negative licensing provides an additional layer of public protection in some jurisdictions, particularly for self-employed practitioners.

The review found diverse literature indicating increasing reliance on and expanding scopes of practice of registered and unregistered health associate professionals^7^—in both HICs [215, 251–254] and LMICs [255–258]. Studies focused on the HPR processes used to support a rationalization of the skills mix and allocation of roles and responsibilities, including education, management and supervision requirements to ensure safe and quality care. The evidence was mixed. Several studies from both HICs and LMICs highlighted safety concerns where role delegation reforms, often involving the administration of medicines, occurred without adequate accompanying measures and supervision and sometimes beyond what was authorized by law. More studies reported positive outcomes, both for program efficiency and patient care. The grey literature yielded extensive evidence of the benefits of skills mix and role delegation reforms, and the ingredients of successful reform initiatives, particularly in dental care, nursing, pharmacy and allied health. There is evidence that negative licensing (where a mandatory code of conduct applies to all unregistered health workers with regulators empowered to investigate breaches and remove unfit workers from the health workforce) provides an additional layer of public protection for health service users [35, 259–265].

#### Regulation of complaints-handling and discipline

**Fig. 13:**
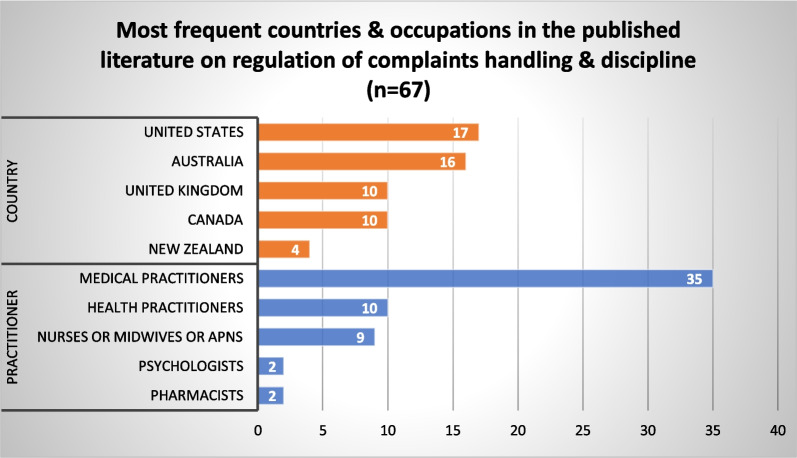
Most frequent countries and health occupations in the published literature on regulation of complaints-handling and discipline

Sixty-seven (67) published articles and 72 grey literature sources included content related to the operation of complaints and disciplinary functions under HPR regimes. The published literature focused primarily on medical practitioners (*n* = 35), followed by health practitioners in general (*n* = 10) and then nurses and midwives (*n* = 9) (Fig. 13). The US was the most prominent jurisdiction (*n* = 17), followed by Australia (*n* = 16), Canada and the UK (*n* = 10 each). Three themes were identified from our synthesis of the published and grey literature.

*First,* there is considerable diversity in the regulatory powers, governance, and processes for managing complaints and discipline, but little evidence on how best to design and deliver effective systems.

Despite the importance of HPR processes for identifying and managing practitioners with conduct, competence, or capacity concerns, there is considerable diversity of arrangements for dealing with complaints and discipline: in the architecture of the disciplinary process, the triggers for regulatory action, the conduct that regulators focus on, the range of powers and penalties available, the extent of monitoring and enforcement activity, the procedural fairness safeguards and the level of transparency and reporting of the performance of these functions [5, 78, 80, 82, 95]. Comparative studies [75, 80, 210, 266–268] were rare, mainly descriptive and primarily of HICs. Three studies addressed challenges with managing complaints and discipline in LMICs [269–271]. Government or regulator commissioned reports in HICs explore some of the systemic complexities and tensions in complaints management, including whether the primary purpose of regulation is punitive or remedial, how these processes fit within broader jurisdictional civil and criminal law and malpractice compensation systems, and how to better support complainants and practitioners throughout the process [2, 3, 53, 58, 272–274]. With a few exceptions, most systems lack transparency, with little evidence of performance reporting or focus on quality improvement.

*Second*, regulators in some HICs are designing risk management and prevention strategies, informed by studies of prevalence rates for disciplinary action.

The literature suggests substantial research efforts in HICs to measure the prevalence rates for disciplinary action in particular cohorts of practitioners and how regulators may use these data to identify and mitigate the risk of harm to the public. A shift to risk-based regulation is evident with disciplinary data analyzed to identify the patterns and characteristics of registrants subject to disciplinary action [41, 42, 275–277]. In the US, multiple studies found that physicians who failed to recertify or allowed their certification to lapse were significantly more likely to be subject to disciplinary action later [172–174, 176]. While several studies examined practitioner stress when subject to disciplinary action [278–280], it is primarily governments and regulators that have commissioned research on the complainant experience [52, 281–285].

*Third*, remediation programs for impaired and poorly performing practitioners and mandatory reporting obligations may be effective public protection mechanisms, albeit with resourcing and implementation challenges.

There is growing interest from regulators and researchers in remediation (returning impaired or poorly performing practitioners to safe and competent practice) and mandatory reporting (legislated obligations on registrants or employers to report certain registrant misbehavior to regulators). Studies have generally reported positive effects of HPR remediation processes, though such programs are resource-intensive [286, 287]. Studies also examined legislated obligations for mandatory reporting as a mechanism for alerting regulators to practitioners or students with conduct, competence or impairment concerns, finding that these obligations may strengthen public protection if carefully structured and clearly communicated [210, 275, 276, 288–290].

#### Regulation of traditional and complementary medicine practitioners

**Fig. 14:**
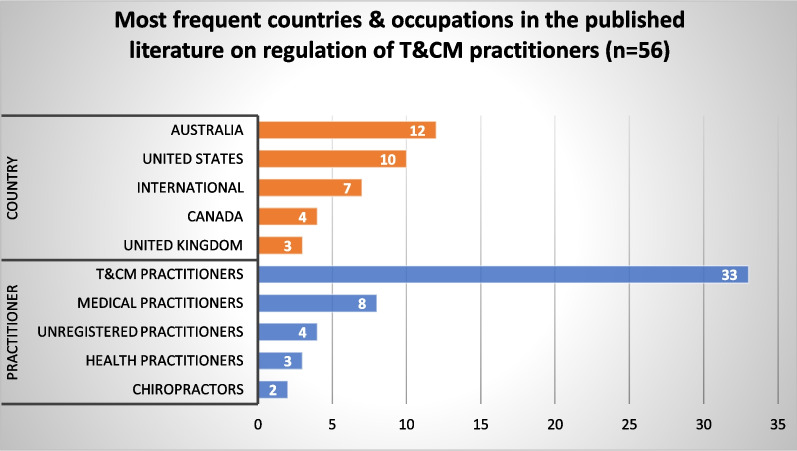
Most frequent countries and health occupations in published literature addressing regulation of T&CM practitioners

There were 56 published articles and 35 grey literature sources relevant to regulating T&CM practitioners. Articles from Australia (*n* = 12), the US (*n* = 10) and international focus (*n* = 7) were prominent (Fig. 14). Three themes were identified from our synthesis of the published and grey literature.

*First*, statutory registration is being extended to more T&CM occupations in more jurisdictions, in response to evidence of risk.

Statutory registration schemes have been enacted at an accelerating rate for T&CM occupations over the past decade, often to preserve Indigenous medicine traditions in LMICs and in response to pressure from representative bodies in HICs [24, 95]. Some jurisdictions have applied regulatory impact assessment processes to inform decisions about whether and how to regulate these occupations [59, 291–293]. These studies suggest the risk profile of some T&CM occupations warrants the level of public protection that statutory registration affords [6, 24, 263, 291, 292, 294–298].

*Second,* statutory registration is a favored strategy of many T&CM professional bodies to prevent entry of untrained practitioners, foster collaborative practice and promote integration into the mainstream healthcare system.

While the literature points to continuing interest in and use of T&CM in LMICs [95, 299–301] and HICs [302–306], studies suggest that T&CM practitioners continue to struggle for institutional recognition of their practice and to engage conventional practitioners in collaborative practice. In LMICs, studies show efforts to better harness Indigenous medicine practitioners to deliver primary care and meet public health goals, with statutory registration a vehicle to elevate the status of Indigenous medicine practitioners and facilitate their integration into mainstream health systems [300, 301, 307, 308]. In HICs, occupational closure is sought to raise standards, protect the public and increase institutional recognition. It may also be pursued to address restrictive regulations that limit practice or prevent access to tools of trade (e.g., herbal medicines).

*Third*, studies suggest that statutory registration works equally well for established and widely practiced T&CM occupations, with some adjustments.

Statutory registration of T&CM occupations has been implemented in both LMICs and HICs. Where such schemes are in operation, studies suggest that this regulatory model works just as well as for other health occupations [263, 291]. A similar range of research concerns was found, such as the content of accreditation standards [309, 310], implementing evidence-based national examinations [24, 311–313], regulatory strengthening [314, 315], and regulating scopes of practice [297, 304, 316–319]. Studies note some of the policy challenges and adjustments required when applying statutory registration to the T&CM occupations, such as evaluating risk, protecting traditional knowledge, applying flexible language requirements, or delivering care to underserved populations [291, 297, 316–318, 320–326].

### Outcomes

#### Impacts of regulation on health workforce and health system outcomes

**Fig. 15:**
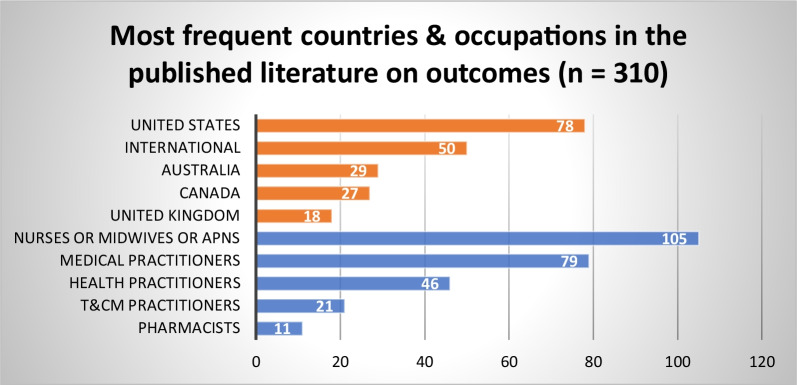
Most frequent countries and health occupations in published literature addressing impacts of HPR on health workforce and health system outcomes

To assess the evidence on the impact of HPR structures and processes in achieving the health workforce and health system outcomes desired by governments and other health system partners, we reviewed studies that reported or measured the following health system and workforce outcomes: safety, quality, capacity/access, capability, effectiveness, quantity (of practitioners), and sustainability. We found 310 empirical studies in the published literature and 105 grey literature sources that discussed one or more of these outcomes when broadly defined. Studies were primarily on nurses, midwives and APN roles (*n* = 105), and medical practitioners (*n* = 79), followed by health practitioners in general (*n* = 46). Like other topics, the US was prominent (*n* = 75), followed by international studies (*n* = 48), Australia (*n* = 29) and Canada (*n* = 27) (Fig. 15). Four themes were identified from the integrated synthesis of the published and grey literature on this topic.

*First*, few jurisdictions have institutionalized arrangements for periodic review and continuous improvement of their HPR systems.

Some literature examined the economic impacts of statutory registration [60, 61, 327–329] and evaluated the effectiveness of a licensing law or the overall performance of a regulator or regulatory system [291, 294, 298, 330–332]. It is difficult to draw conclusions from these studies given the diversity of topics covered. Findings often included calls for stronger regulation, expansion of statutory registration to more occupational groups, and greater accountability to operate in the public interest. In a small number of Anglophone HICs, extensive grey literature shows regulatory reform efforts over several decades to strengthen governance, transparency and government oversight and expand and codify statutory powers and functions [2, 4, 53, 55, 58, 333–336]. Unscheduled or one-off regulatory reviews led to significant legislative and administrative reform, generally in response to a crisis or regulatory failure [2, 337–339]. The UK, New Zealand, and Ontario (Canada) were identified as having a proactive system of periodic review of the performance of regulators. An active program of continuous improvement was evident in the UK with the operation of its meta-regulator, the Professional Standards Authority, and in New Zealand, a requirement for independent performance reviews of regulatory authorities has been legislated. American bodies such as the National Council of State Boards of Nursing and the Federation of State Medical Boards also featured in the grey literature on regulatory system improvement, as did international organizations, including the OECD and the WHO [9, 29, 108, 314, 340–346].

*Second*, further evaluation is needed of alternative models for regulating the health workforce, such as negative licensing and quality assured voluntary registers.

We identified studies in the published literature that addressed the effectiveness of other forms of occupational regulation, such as voluntary certification [6, 82, 296, 306, 347–349] and negative licensing [6, 32, 263, 264, 295, 296, 350]. In a few of these studies, researchers were critical of non-statutory certification or negative licensing schemes, instead advocating for the level of public protection afforded by statutory registration/licensing. The grey literature search found government-commissioned studies that examined the costs and benefits of different approaches to HPR in achieving the government public protection objectives [35, 292, 293, 351–354].

*Third*, regulatory strengthening initiatives in LMICs aim to build stronger regulatory institutions, infrastructure, networks and governance, with some evidence of success.

The review identified studies that evaluated the impacts of HPR system strengthening initiatives, mainly in LMICs (sub-Saharan African countries of Uganda, Nigeria, Kenya, Eswatini, Malawi and South-East Asian countries of Cambodia and Vietnam) [64–67]. These studies suggest that the Regulatory Function Framework developed through the African Health Profession Regulatory Collaborative program is a valuable tool for designing and implementing HPR strengthening projects and evaluating the effectiveness of system strengthening initiatives in LMICs.

*Fourth,* studies that compare regulatory regimes across multiple jurisdictions were mostly descriptive, underscoring the need for more robust outcome measures and measurement tools.

Academic and grey literature sources that compared the operation of HPR schemes across multiple jurisdictions or globally were mostly descriptive, comparing key features such as the scope and governance of schemes or specific regulatory functions, sometimes including a historical perspective [5, 24, 78, 95, 343]. Some studies evaluated specific regulatory interventions, such as NLEs [77], mandated CPD [135], maintenance of certification schemes [175], processes for dealing with misconduct [267], mandatory reporting obligations [288], and the application of administrative sanctions [80]. Academic and grey literature provide frameworks for comparative studies of HPR regimes that can be used to strengthen methodologies and standardize outcome measurement [23, 89, 90].

## Discussion

This review aimed to assess the evidence base on HPR design and delivery in achieving health system goals and supporting health workforce availability, accessibility, quality, and sustainability. Through our evidence synthesis, we identified several key themes that were categorized by HPR structures, processes, and outcomes.

Certain governance trends, such as multi-practitioner regulators or umbrella laws, were evident, but the lack of standardized typology complicated comparisons of these governance arrangements across jurisdictions and occupations. Some jurisdictions have regulatory management systems that embed evidence-informed regulatory policymaking, particularly when deciding changes to the scope of a licensing scheme or introducing new practice restrictions. These systems are designed to better target regulation and ensure legislative frameworks are regularly reviewed and fit for purpose. Some regulators use risk-based regulation tools, weighing risk to the public with the need for access to health services. More jurisdictions are undertaking period review and reform to maintain a fit-for-purpose regulatory framework.

Most studies in this review focused on statutory registration schemes, and evidence suggested this model of HPR is increasingly being enacted across various jurisdictions and practitioner groups. The review found evidence suggesting this HPR model may strengthen public protection for some T&CM occupations based on risk profiles. For associate health professionals, lower-cost models of quality assurance (for example, non-legislated certification schemes, co-regulation,^8^ or negative licensing) may be sufficient, but further study of these models is required.

HPR generally has been challenged to keep pace with the demands for greater flexibility arising from collaborative team-based models of care and a more dynamic division of labor in health care. This tension is most apparent in the literature on scope of practice regulation. While necessary to maintain a flexible, responsive and sustainable health workforce, scope of practice reforms are among the most highly charged policy issues facing legislators and health care regulators [229, 355]. There are costs to the health system, the health workforce and health consumers when scopes of practice are too tightly regulated in a way that is unresponsive to reform. Prescribing rights are a case in point, with conflicts over prescribing authority often reflecting competing interests between occupations. Such tensions suggest demarcation disputes between occupational groups should be managed by government in a more interventionist way to expedite reform. The urgency created by the COVID-19 pandemic highlighted how quickly scope of practice reforms can be enacted. These reforms require systematic evaluation.

Evidence from LMICs and HICs suggests that HPR can contribute to workforce planning, development, supply and distribution. For many governments, the capacity to carry out accurate and effective workforce planning is hampered by a lack of health workforce data. This gap could potentially be bridged by leveraging HPR registry data. This generally requires a clear legislative basis that authorizes regulators to collect this data and robust information technology systems to provide it in a de-identified form to health system partners such as governments, educators and researchers.

The evidence in our review suggests that widespread barriers impact the mobility of practitioners, despite considerable efforts to standardize and harmonize regulatory arrangements across jurisdictions. Mutual recognition schemes create incentives to streamline qualification recognition and registration processes for IEHPs [356], but implementation has been variable.

The review also identified evidence supporting the impact of outcomes-based CPD models on continuing competence to practice and patient safety. Limited evidence suggests CPD may be valuable in upskilling specific health occupational groups in LMICs if delivered as part of a broader workforce development strategy. While revalidation mechanisms have been considered and implemented in a few cases, the resource-intensive nature of these schemes means the uptake has been limited and is unlikely to be considered or implemented in LMICs. Beyond making CPD mandatory for registration renewal, applying other risk-based strategies that target continuing competence requirements to higher-risk groups may be more cost-effective.

Reform in HPR was evident in many contexts. Throughout sub-Saharan Africa, South-East Asia and in Mekong countries (Cambodia, Laos, Vietnam), statutory registration schemes are a relatively recent development, with regulatory models, governance and institutions being adapted to local circumstances. In Africa, the Caribbean and the Pacific Island countries, networks of regulators are working together to set standards for education and training, develop CPD programs, and support health system strengthening. In many countries, statutory registration schemes have been introduced to accelerate the integration of indigenous medicine and T&CM practitioners and to enable the recruitment of this workforce to better address public health priorities.

A few alternative models of occupational regulation were found that target the unregistered workforce and provide a lower-cost alternative to statutory registration for lower-risk health occupations. The accredited registers program in the UK (and more recently in Hong Kong) and negative licensing/prohibition order powers in Australia and the USA (Minnesota) are notable examples. Innovation is also evident in some Anglophone HICs where statutory registration schemes have been operating for over a century.

Four areas of regulatory innovation we identified in the literature are worth noting. First, regulators are applying the tools of risk-based regulation, using data analytics to identify risk hotspots and design targeted and time-limited strategies to prevent or minimize harm. Second, there is more focus on health system linkages and quality assurance networks, including cooperative efforts between regulators, government, non-government standard-setting agencies, and other organizations. Third, more jurisdictions are applying good regulatory practices for evidence-informed policy decisions about extending regulation to specific occupations, designing HPR legislation, and developing standards that impact practice and competition within the health market. Lastly, regulators' mandates in some countries now include a broader role in health system improvement, extending beyond public protection to societal objectives such as reducing inequality and increasing diversity. This requires greater accountability and transparency of regulation and regulators, and governance structures that support a partnership between government, regulators, practitioners, health care consumers and civil society.

## Limitations of the review

A critical limitation of comparative HPR research and synthesizing the state of HPR evidence is the lack of standardized language. Definitional ambiguity arises from how terms such as self-regulation, registration, licensing, and accreditation are used differently in different countries and contexts [78, 83]. This lack of standard language made comparative analysis and synthesis difficult, given the diversity of PICO (populations, interventions, contexts, outcomes) elements in studies and the wide variety of research designs. While we used rigorous extraction and thematic analysis processes to strengthen our review, the largely descriptive nature of the underlying evidence made it challenging to link regulatory interventions to outcomes of interest and to draw causal inferences. More consistent definitions would enhance the global understanding of HPR, improve the design of regulatory regimes and the mobility of practitioners, and ultimately increase public safety and access to health care [357].

Publications from the US, UK, Australia and Canada dominate the literature. This is typical of systematic reviews and partly reflects an artifact of funding availability and the broader research landscape. As a result, the themes and findings strongly reflect matters of interest and contention in these high-income Anglophone countries. In the design of the review (the framing of the research questions, topics and inclusion criteria) and the synthesis and presentation of the findings, we have highlighted available data from LMICs and discussed the implications of our findings for lower-resourced environments.

A further limitation is that the literature searches were conducted in 2021 and thus more recent sources are not included in this review. Although the count of studies by topic would be altered, we do not anticipate that these new studies would have a substantial impact on the overall findings.

## Key evidence gaps for future research

We identified areas where critical knowledge gaps remain. As noted, there is less published literature on HPR structures, processes, and outcomes in LMICs. Evaluations should focus on identifying the highest impact HPR structures and processes and viable alternatives to full statutory registration schemes, such as negative licensing, particularly for lower-risk occupational groups.

There were few studies in the published literature that had a robust measurement of the outcomes of regulatory interventions on patient safety or quality of care or that systematically measured whether a regulatory system was effective in achieving its objectives. Evaluating different institutional and governance arrangements against a standardized framework would enable stronger cross-jurisdictional comparisons of HPR performance. For instance, comparative studies of the performance of regulatory regimes against outcome measures such as safety and quality of care, health workforce availability and distribution, cost-effectiveness, or against process criteria, such as accountability transparency, and agility could increase our understanding of what works. Schemes that lack basic transparency measures, such as online searchable registers, online patient complaint submission, and published disciplinary decisions, may not make the best use of regulatory data for health system improvement. Also, despite an increasing focus on risk-based approaches to HPR, robust evaluations of the impact of these approaches on patient safety and health workforce quality are required.

Knowledge gaps remain around the relative benefits of national licensing examinations and HPE accreditation in assuring the quality of the health workforce. Despite increased research around remediation programs and mandatory reporting obligations, more evidence is required on the effectiveness of these specific HPR complaints and discipline processes across jurisdictions, HPR models, and occupational groups.

The COVID-19 pandemic has highlighted the importance of agile HPR processes and effective linkages between HPR and health system partners. Empirical studies of the effectiveness of HPR pandemic responses have continued to be published after our review’s inclusion dates [358–360]. Further research in this area would help evaluate HPR reforms and innovations to determine which changes should be maintained long-term and which would be most beneficial for future crises. This research should also assess the effectiveness of system linkages and how HPR is best placed to contribute to emergency responses that require a fit-for-purpose surge workforce.

## Conclusion

This paper provides a comprehensive review of the existing literature on HPR, synthesizing evidence from a broad range of academic and grey sources. The findings were categorized into key themes based on our conceptual framework encompassing the structures, processes, and outcomes of HPR.

Under structures, we examined regulatory governance systems, regulatory institutions, and system linkages. Processes included registration and monitoring of practitioners' continuing competence, accreditation of entry-to-practice education programs, regulation of scopes of practice, management of complaints and discipline, and regulation of T&CM practitioners. Outcomes focused on the impact of HPR structures and processes on health system and workforce outcomes.

The findings of the review are summarized into key messages and themes for each topic. Under *structures*, governance reforms in HPR demonstrated trends towards multi-profession regulators, enhanced accountability, and risk-based regulatory principles, though comparisons between HPR models were complicated by a lack of a standardized HPR typology. HPR plays a key role in supporting government workforce strategies, despite persisting challenges in cross-border recognition of qualifications and portability of registration. Under *processes*, scope of practice regulation needs to adapt to modern health system environments, and these reforms can enhance access and quality. Under *outcomes*, alternatives to statutory registration for lower-risk health occupations can enhance health service quality and consumer protection, while a systematic approach to evaluating regulatory failures and standardizing evaluation frameworks can aid regulatory strengthening. Knowledge gaps remain around the outcomes and effectiveness of specific HPR processes, including continuing professional development models, national licensing examinations, accreditation of health practitioner education programs, remediation programs, mandatory reporting obligations, and statutory registration of traditional and complementary medicine practitioners.

Policymakers, governments, and regulators can use these insights to inform regulatory design and practice. It is important to consider the limitations and gaps in the available evidence, including the dominance of high-income Anglophone countries and descriptive studies in the reviewed literature. These limitations and gaps warrant caution when interpreting and applying these findings across different jurisdictions and professions.

To address these gaps, we recommend prioritizing further research on regulatory outcomes. Both research funders and governments should invest in generating more outcomes-based evidence to inform regulatory design and reform efforts. Additionally, a systematic approach should be adopted to track and evaluate the effectiveness of regulatory interventions and innovations in achieving health workforce and health systems goals.

### Supplementary Information

**Additional file 1.** Research design, search strategy, and modified PICO framework.

**Additional file 2.** Countries and health occupations of focus in all included published articles.

**Additional file 3.** Additional information on included published and grey literature sources.

## Data Availability

Data analyzed during this study are included in this published article and its supplementary information files. Additional details are available in the full review report and appendices prepared for the World Health Organization.

## Footnotes

1 T&CM was the term commonly used in the literature we reviewed. T&CM is part of a broader category of traditional, complementary and integrative medicine, the term used by the WHO.

2 See also our protocol that was registered at Open Science Framework. (https://doi.org/10.17605/OSF.IO/EMWSU).

3 Note that the articles classified as “international” in this review were broad in scope. Rather than focusing on a single country, group of countries or region, these articles generally took a broad look at an HPR issue or topic, such as a scoping review of all English language literature on a HPR topic. If an article focused on a group of LMICs, it was classified as LMIC (a separate category in our data extraction) rather than international.

4 Examples of mutual recognition agreements include ASEAN in South-East Asia, CARICOM, Europe, Trans-Tasman Mutual Recognition Arrangement, Licensure Compacts in the US and the previous North American Free Trade Agreement.

5 Since 2010, statutory registration schemes have been established or extended in jurisdictions as diverse as Australia, Brazil, Burkina Faso, Cambodia, Canada, Cook Islands, Czech Republic, Fiji, India, Malaysia, Mali, New Zealand, Pakistan, Senegal, Singapore, the Philippines, Samoa, Uganda, the UK and the USA. Note this is not an exhaustive list.

6 We use NLE here to describe a large-scale examination either provided or commissioned by a health practitioner regulator and used to determine whether an applicant is qualified to practice. We use this term to encompass examinations at the national level and at the sub-national level in federated systems of government.

7 These practitioners are classified as health associate professionals under the ILO classification. However, the terms assistant and support workforces were often used in the literature.

8 Co-regulation involves a self-regulating professional association with certification functions that are either delegated from or recognized by government. This government recognition or delegation may be conditional on the certification body meeting specified standards. This recognition process establishes, in effect, a partnership between government and the certifying body. The benefits that flow to practitioners from certification create incentives for practitioners to comply with the professional association’s standards.
