# Supplementary material for: Design, delivery and effectiveness of health practitioner regulation systems: an integrative review
Source: Hum Resour Health. 2023 Sep 4;21:72. doi: 10.1186/s12960-023-00848-y (PMC10478314; doi:10.1186/s12960-023-00848-y)
Supplement: Supplementary file 1 — Additional file 1. Research design, search strategy, and modified PICO framework. [file 12960_2023_848_MOESM1_ESM.docx]

# Additional File 1

***Modified rapid integrative review design***


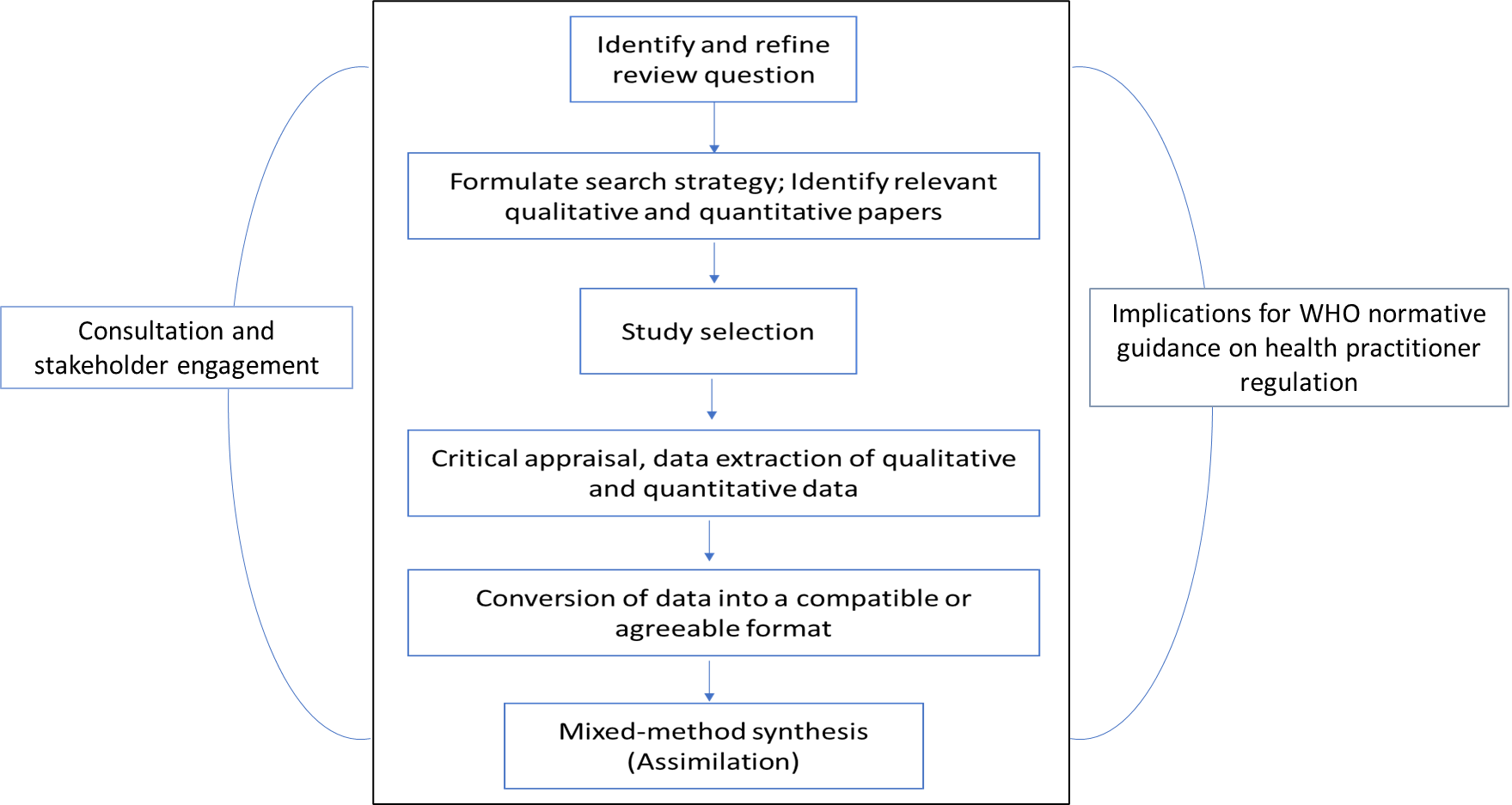


***Examples of academic database search strategies***

##### MEDLINE via OVID conducted on 1 September 2021

| **Search** | **Query** | **Records Retrieved** |
| --- | --- | --- |
| 1 | (acupuncturist* or birth attendant* or clinician* or dental assistant* or dental auxiliar* or dental hygienist* or dentist* or denturist* or dieti?ian* or doctor* or emergency medical technician* or healer* or home health aide* or lab* personnel* or lab* worker* or lactation consultant* or masseuse* or midwife or midwives or naturopath* or nurse* or nutritionist* or optician* or optometrist* or orthoptist* or osteopath* or paramedic or paramedics or physician* or physiotherapist* or podiatrist* or prosthetist* or psychotherapist* or radiographer* or sonographer* or surgeon*).tw. | 1,317,317 |
| 2 | ((health or health care or healthcare) adj1 (professional or professionals or personnel or practitioner* or worker*)).tw. | 133,303 |
| 3 | (therapist adj1 (acupunctur* or alternative or complementary or integrative or investigative or investigational or massage or medical or mind body or "mind and body" or musculoskeletal or natural or occupational or physical or reflex* or sensory or spiritual)).tw. | 4,463 |
| 4 | Allied Health Personnel/ or Allied Health Occupations/ or Anesthetists/ or Audiologists/ or Community Health Workers/ or exp Dental Auxiliaries/ or exp Dental Staff/ or exp Dentists/ or Emergency Medical Technicians/ or Health Occupations/ or Health Workforce/ or Home Health Aides/ or Infection Control Practitioners/ or Medical Laboratory Personnel/ or exp Medical Staff/ or exp Nurses/ or exp Nursing Assistants/ or exp Nursing Staff/ or Nutritionists/ or Occupational Therapists/ or Operating Room Technicians/ or Optometrists/ or Pharmacists/ or Pharmacy Technicians/ or Physical Therapy Assistants/ or exp Physician Assistants/ or Physicians/ or Psychotherapists/ | 368,003 |
| 5 | 1 or 2 or 3 or 4 | 1,593,487 |
| 6 | (ban or banned or banning or certification* or certify or certified or credential* or coregulation or deregulation or governance or license or licensed or licensing or licensure or professional standard* or prohibit* or regulation or regulatory or regulations).tw. | 1,371,757 |
| 7 | exp Certification/ or Jurisprudence/ or Legislation, Medical/ or Legislation, Nursing/ or Legislation, Pharmacy/ or Licensure/ or Licensure, Dental/ or Licensure, Medical/ or Licensure, Nursing/ or Licensure, Pharmacy/ or Health Policy/ or Credentialing/ | 155,553 |
| 8 | 6 or 7 | 1,503,388 |
| 9 | ((availab* or capabilit* or capable or competen* or effectiv* or efficiency or efficient or quality or safety) adj2 (clinician* or personal or personnel or professional* or worker* or workforce*)).tw. | 16,763 |
| 10 | Efficiency/ or Quality Improvement/ or Quality Indicators, Health Care/ or Health Services Accessibility/ or Health Equity/ or Clinical Competence/ or Professional Competence/ or Professional Autonomy/ or Patient Safety/ | 284,276 |
| 11 | 9 or 10 | 298,079 |
| 12 | 5 and 8 and 11 | 11,154 |
| 13 | limit 12 to (chinese or czech or dutch or english or finnish or french or portuguese or russian or spanish or swedish) | 10,886 |
| 14 | limit 13 to yr="2010 -Current" | 5,632 |

##### EMBASE via OVID conducted on 1 September 2021

| **Search** | **Query** | **Records Retrieved** |
| --- | --- | --- |
| 1 | (acupuncturist* or birth attendant* or clinician* or dental assistant* or dental auxiliar* or dental hygienist* or dentist* or denturist* or dieti?ian* or doctor* or emergency medical technician* or healer* or home health aide* or lab* personnel* or lab* worker* or lactation consultant* or masseuse* or midwife or midwives or naturopath* or nurse* or nutritionist* or optician* or optometrist* or orthoptist* or osteopath* or paramedic or paramedics or physician* or physiotherapist* or podiatrist* or prosthetist* or psychotherapist* or radiographer* or sonographer* or surgeon*).tw. | 1,850,256 |
| 2 | ((health or health care or healthcare) adj1 (professional or professionals or personnel or practitioner* or worker*)).tw. | 175,029 |
| 3 | (therapist adj1 (acupunctur* or alternative or complementary or integrative or investigative or investigational or massage or medical or mind body or "mind and body" or musculoskeletal or natural or occupational or physical or reflex* or sensory or spiritual)).tw. | 7,636 |
| 4 | exp advanced practice provider/ or acupuncturist/ or exp anesthetist/ or audiologist/ or care coordinator/ or childbirth educator/ or clinical laboratory personnel/ or clinician/ or exp dental personnel/ or diabetes educator/ or dietitian/ or exp eye care professional/ or foreign physician/ or health auxiliary/ or health care personnel/ or health practitioner/ or health workforce/ or hospital personnel/ or hospital pharmacist/ or hospital physician/ or lactation consultant/ or exp manual therapist/ or medical staff/ or mental health care personnel/ or exp midwife/ or exp nurse/ or nursing assistant/ or nursing home personnel/ or nursing staff/ or occupational therapist/ or occupational therapy assistant/ or operating room personnel/ or ophthalmic technologist/ or orthotist/ or paramedical personnel/ or paramedical profession/ or perfusionist/ or exp pharmacist/ or pharmacy technician/ or physician/ or physiotherapist assistant/ or physiotherapist/ or exp prosthetist/ or podiatrist/ or psychiatrist/ or exp radiographer/ or rescue personnel/ or respiratory therapist/ or Shaman/ or sonographer/ or speech language pathologist/ or surgeon/ or traditional birth attendant/ or traditional healer/ | 1,239,398 |
| 5 | 1 or 2 or 3 or 4 | 2,517,164 |
| 6 | (ban or banned or banning or certification* or certify or certified or credential* or coregulation or deregulation or governance or license or licensed or licensing or licensure or professional standard* or prohibit* or regulation or regulatory or regulations).tw. | 1,722,001 |
| 7 | accreditation/ or certification/ or health care policy/ or license/ or licensing/ or medicolegal aspect/ or recertification/ | 323,380 |
| 8 | 6 or 7 | 1,995,595 |
| 9 | ((availab* or capabilit* or capable or competen* or effectiv* or efficiency or efficient or quality or safety) adj2 (clinician* or personal or personnel or professional* or worker* or workforce*)).tw. | 22,230 |
| 10 | clinical effectiveness/ or clinical competence/ or health equity/ or health care access/ or health care quality/ or productivity/ or total quality management/ or patient safety/ or professional competence/ or professional practice/ | 786,221 |
| 11 | 9 or 10 | 803,275 |
| 12 | 5 and 8 and 11 | 33,193 |
| 13 | limit 12 to (chinese or dutch or english or finnish or french or portuguese or russian or spanish or swedish) | 32,432 |
| 14 | limit 13 to yr="2010 -Current" | 18,835 |

***Modified PICO Framework for Framing HPR Research Questions***

| **Modified PICO component** | **Description** |
| --- | --- |
| **P**opulation | Health practitioners  *(including medical practitioners, nurses and midwives, pharmacists, dental, allied health, traditional and complementary medicine, community health workers, epidemiologists, other registered and unregistered health workers; internationally qualified/foreign trained practitioners)* |
| **I**ntervention | Approaches to health practitioner regulation  *(regulatory models, powers, tools, systems – for example, profession-led regulation, self-regulation, co-regulation, statutory regulation, negative licensing)* |
| **C**ontext | Countries/regions  *(with diverse linguistic, political, social, economic and legal systems; for example common law, civil code, Islamic law, customary law, mixed or hybrid systems; basic, emerging and mature health systems)* |
| **O**utcome | Goals of health practitioner regulation  *(for example safety, quality, capability, quantity, effectiveness and sustainability)* |

Note: A traditional PICO framework delineates ‘C’ as Control, but we knew that for this review on HPR, research designs that include a control group would be highly unlikely. Thus, the research team decided to capture important contextual country information, which was deemed highly relevant.
