# Supplementary material for: Design, delivery and effectiveness of health practitioner regulation systems: an integrative review
Source: Hum Resour Health. 2023 Sep 4;21:72. doi: 10.1186/s12960-023-00848-y (PMC10478314; doi:10.1186/s12960-023-00848-y)
Supplement: Supplementary file 2 — Additional file 2. Countries and health occupations of focus in all included published articles. [file 12960_2023_848_MOESM2_ESM.docx]

# Additional File 2

***Country of focus in all included published articles (n=410)***

| **Country** | **Number of Articles** |
| --- | --- |
| United States | 107 |
| International | 65 |
| Australia | 39 |
| Canada | 35 |
| United Kingdom | 34 |
| European Union | 13 |
| India & Sub-Saharan Africa | 11 |
| New Zealand, Uganda | 7 |
| Ireland | 6 |
| Ghana, Nepal, South Africa, South Korea | 5 |
| Cambodia, Kenya | 4 |
| Brazil, China, East, Central, and Southern Africa, Ethiopia, Hong Kong, LMIC, Malawi, The Netherlands, Pakistan, Portugal, Tanzania | 3 |
| ASEAN member states, Asia and Western Pacific, Bangladesh, Eswatini, Germany, Georgia, Indonesia, Japan, Nigeria, Philippines, Sweden, Thailand, Vietnam | 2 |
| Afghanistan, Bhutan, Burkina Faso, Cambodia, Czech Republic, Finland, Guinea-Bissau, Iran, Israel, Italy, Lao People's Democratic Republic, Latin America and the Caribbean, Lesotho, Malaysia, Mali, Mongolia, Mozambique, Myanmar, North America, Norway and Denmark, Singapore, South East Asia region, Spain, Sri Lanka, Sudan, Taiwan, UAE | 1 |

***Health occupations of focus in all included published articles (n=410)***

| **Practitioner** | **Number of Articles** |
| --- | --- |
| medical practitioners | 102 |
| health practitioners | 64 |
| nurses | 45 |
| nurse practitioners/APN | 43 |
| T&CM practitioners | 33 |
| nurses and midwives | 26 |
| pharmacists | 14 |
| dentists | 12 |
| unregistered health practitioners | 11 |
| midwives | 9 |
| internationally educated medical practitioners, physician assistants | 8 |
| psychologists | 6 |
| paramedics/EMT | 5 |
| dental hygienists, non-medical practitioners | 4 |
| Chiropractors, community health workers, internationally educated nurses | 3 |
| internationally educated health practitioners, nutritionists, occupational therapists, osteopaths physiotherapy, radiographers, support workers, T&CM users | 2 |
| Aboriginal & Torres Strait Islander Health workers/practitioners, behavioral health practitioners, dental technicians, Health service users, home care aides, Indigenous healers, Non-professional public directors, nursing assistants, occupational therapist assistant and physiotherapist assistant, pharmacy technicians, social workers, traditional birth attendants (TBAs) and skilled birth attendants (SBAs) | 1 |
